# Extracellular Vesicles: A Mailcoach From Mast Cell to Other Cell Species

**DOI:** 10.1002/iid3.70452

**Published:** 2026-04-23

**Authors:** Bingqi Zhang, Yueshan Sun, Tao Jiang, Runmin Long, Yuanbiao Guo

**Affiliations:** ^1^ Department of Clinical Laboratory, The Eighth People's Hospital of Chengdu Geriatric Hospital of Chengdu Medical College Chengdu Sichuan China; ^2^ Medical Research Center, The Affiliated Hospital of Southwest Jiaotong University The Third People's Hospital of Chengdu Chengdu Sichuan China; ^3^ Department of Clinical Laboratory The People's Hospital of Jianyang City Jianyang Sichuan China

**Keywords:** dendritic cells, epithelial cell, extracellular vesicles, macrophage, mast cells, T cells, tumor cells

## Abstract

**Background:**

Mast cells (MCs) are pivotal immune cells. Beyond soluble mediators, mast cell‐derived extracellular vesicles (MC‐EVs) are crucial for intercellular communication. MC‐EV heterogeneity, influenced by cellular activation and microenvironment, drives diverse biological roles. This review synthesizes current MC‐EV biology, cellular interactions, and translational potential in immunity, inflammation, and disease.

**Methods:**

We systematically reviewed literature (2001–2024) from PubMed, Web of Science, and Scopus on MC‐EV biogenesis, cargo, and functional roles from in vitro, in vivo, and clinical studies, emphasizing recent advances across diverse disease context.

**Results:**

MC‐EVs exhibit profound heterogeneity, with distinct cargo profiles driven by cellular activation (e.g., inflammatory proteins/miRNAs from activated MCs vs. homeostatic cargoes from resting MCs). These vesicles critically mediate intercellular communication, enhancing immune responses by promoting dendritic cell maturation, T lymphocyte activation, and IL‐5 production in group 2 innate lymphoid cells. Their influence extends to epithelial cells, regulating epithelial‐to‐mesenchymal transition and disrupting barrier function context‐dependently. Pathologically, MC‐EVs exert dual roles in cancer, drive organ damage in mastocytosis, and modulate tissue microenvironments (e.g., neuroinflammation, bone marrow). This diverse functionality highlights their potential as diagnostic biomarkers and engineered therapeutic tools.

**Conclusions:**

MC‐EVs are critical, multifaceted mediators orchestrating communication between mast cells and diverse cell types, and mediate context‐specific immune modulation, remodeling, and disease progression. Deciphering cargo sorting, release and recipient uptake will enable MC‐EV–based biomarkers and therapies for inflammatory, neoplastic, and hematopoietic conditions.

## 1 Introduction

Mast cells (MCs), unique tissue‐resident immune cells, exhibit heterogeneity in their granule composition and transcriptomes [1, 2]. This diversity leads to the identification of distinct subsets across various tissues, primarily divided into two main categories: tryptase‐rich MCs found in mucosa (MCT), and connective tissue (MCTC) rich in both tryptase and chymase [3]. Their heterogeneity, growth, differentiation and maturation are influenced by interactions with resident stromal cells and the local anatomical niches [4], and extends to the extracellular vesicles (EVs) they release. Importantly, EVs are not exclusive to MCs but form a heterogeneous population derived from diverse cellular sources in the tissue microenvironment, including mesenchymal stem cells (MSCs), fibroblasts, immune cells, and tumor cells. This multi‐cellular origin introduces functional diversity in cargo composition (e.g., proteins, lipids, and miRNAs), complicating intercellular signaling networks and necessitating source‐specific characterization for therapeutic applications [5, 6, 7].

As gatekeepers in inflammation and immune responses, MCs help maintain homeostasis and participate in pathogen defense, wound healing, vascularization, tissue remodeling, fibrosis, and autoimmune disease [2, 8]. It's widely recognized that MCs act as facilitators of communication between the innate and adaptive immune systems by interacting with various immune cells, such as T cells, B cells, macrophages, and dendritic cells, to regulate immune responses and maintain physiological equilibrium [9], in part through EV‐mediated signaling mechanisms The role MCs in tumors is complex, involving processes such as the release of pro‐angiogenic factors within the tumor microenvironment [10]. However, in the case of nasopharyngeal cancer, a specific subset of MCs with high expression of tumor necrosis factor (TNF) but low levels of vascular endothelial growth factor A (VEGFA) has been associated with improved clinical outcomes [11]. These interactions, whether over short or long distances within the body, are increasingly recognized to be mediated, at least in part, by EVs, which serve as key carriers of intercellular signaling and functional heterogeneity [12] [13, 14].

Therefore, understanding how MC‐derived EV heterogeneity integrates with EVs from other cellular sources is critical for deciphering their roles in complex tissue microenvironments. In this review, we explore how MC‐EVs regulate interactions with immune, tumor, and stromal cells, and discuss their implications for disease mechanisms, vaccine development, and targeted biological therapies.

### 1.1 Reorganization of the Features and Biological Functions of Mc‐Evs

MCs monitor and respond to changes in metabolism and immune status in their surrounding microenvironment. When stimulated, particularly during allergic responses, they undergo degranulation and release various inflammatory mediators, including tryptase, carboxypeptidase, histamine, and interleukin [15]. In recent years, studies have demonstrated that MCs can produce EVs not only in the activated/stimulated state (Active‐MC‐EVs), but also in the resting state (Rest‐MC‐EVs). Importantly, MC‐derived EVs represent only one component of a broader and heterogeneous EV population within tissues, where EVs released from multiple cell types coexist and interact, thereby contributing to the complexity of intercellular communication.

These MC‐EVs differ in terms of quantity, membrane proteins, constitutive lipids, luminal RNAs and proteins, and their respective functions (Table 1 Differences of MC‐EVs in resting and activated state of MCs).

**Table 1:** Differences of MC‐EVs in resting and activated state of MCs.

Project	Rest‐MC‐EVs	Active‐MC‐EVs	Mast cell type	Activators	References
Amount	less	more	BMMC	DNP‐ IgE	[16]
			PMC and SpMC		[17]
			Human synovium MC	human myeloma IgE	[18]
MC‐specific proteins*	less	more	Human synovium MC	human myeloma IgE	[18]
MC‐associated protein#	less	more	BMMC	DNP‐ IgE	[16]
EV‐surface markers	CD9	CD63	PMC and SpMC	DNP‐ IgE	[17]
Lipid cholesterol	PA(30:1), PC(36:1) and SM(42:3)	PE(32:1), PI(36:2), BMP(40:6)	PMC and SpMC	DNP‐ IgE	[17]
Biological functions of enriching proteins	Glycolytic process, positive regulation of cell adhesion and cell migration	Redox reaction, lipid metabolic processes, signal peptide processing, and vesicle‐mediated transport	BMMC	DNP‐ IgE	[16]
lncRNAs	H19	Zfas1, Snhg20, Jpx, Snhg4, Neat1, Malat1	BMMC	DNP‐ IgE	[16]
Average length of microRNA	> 60nt	< 40nt	BMMC	DNP‐ IgE	[16]
microRNAs	miR‐142a‐5p, miR‐350‐5p, miR‐29a‐5p和miR‐700‐3p	miR‐ 126a‐3p, miR‐21a‐3p, miR‐210‐3p和miR‐150‐5p	BMMC	DNP‐ IgE	[16]
	/	miR23b‐3p, miR103a‐3p	Human synovium MC	human myeloma IgE	[18]
	miR‐7022‐5p, miR‐532‐5p, etc.	miR‐409‐3p, miR7234‐5p, etc.	Murine P815	LPS	[19]

Note:*FcεRI and KIT, #tryptase, carboxypeptidase A, and IL‐4.

Abbreviations: BMP, bis(monoacylglycero) phosphate; BMMC, Bone Marrow Mesenchymal Stem Cells; DNP‐IgE, DNP‐specific IgE; LPS, Lipopolysaccharide; PA, phosphatidic acid; PC, phosphatidylcholine; PE, phosphatidylethanolamine; PI, phosphatidylinositol; PMC, peritoneal MC; PS, phosphatidylserine; SM, sphingomyelin; SpMC, spleen‐derived MC.

Most research consistently indicates that activated MCs release a significantly higher quantity of EVs compared to their resting counterparts [16] [17, 18]. However, discrepancies exist regarding the particle size distribution of Rest‐MC‐EVs versus Active‐MC‐EVs. For instance, Groot Kormelink et al. demonstrated that IgE/anti‐IgE‐stimulated connective tissue MCs (CTMCs) and mucosal MCs (MCTs) produced Rest‐MC‐EVs with broader size heterogeneity compared to Active‐MC‐EVs. In contrast, studies on bone marrow‐derived MCs (BMMCs) reported no significant differences in EV size between resting and activated states [16, 18]. These inconsistencies may reflect the intrinsic heterogeneity of EV populations, influenced by cellular origin, activation state, and microenvironmental context.

The lipid composition of EVs reflects the activation state and phenotypic characteristics of their parent MCs [17]. Notably, MCs express the stem cell factor (SCF) receptor KIT and the high‐affinity IgE receptor (FcεRI), which trigger activation and degranulation upon stimulation [20, 21]. Given the essential role of several phospholipids in vesicle formation and stabilization, it is plausible that these lipids may influence Active‐MC‐EVs secretion [22, 23].

Functionally, MC‐EVs hold higher levels of FcεRI and KIT in activated states, along with EV marker protein CD63 [18, 24], suggesting their association with MC activation and vesicle origin [17]. Correspondingly, Active‐MC‐EVs contain abundant inflammatory factors and proteins involved in redox reaction, lipid metabolism, and vesicle‐mediated transport. Correspondingly, Active‐MC‐EVs contain abundant inflammatory factors and the proteins involved with redox reaction, lipid metabolic processes, signal peptide processing, and vesicle‐mediated transport. In contrast, Rest‐MC‐EVs tend to be enriched in proteins associated with cellular homeostasis, metabolic processes, and intercellular adhesion, suggesting a role in maintaining tissue equilibrium rather than promoting inflammatory activation.

The RNA cargo of MC‐derived EVs exhibits subtype‐specific enrichment patterns. Rest‐MC‐EVs are enriched in lncRNAs and longer miRNA isoforms, whereas Active‐MC‐EVs preferentially package mature miRNAs associated with immune activation, such as miR‐142‐3p [16] [25]. This dynamic remodeling of EV cargo reflects stimulus‐dependent regulation and represents a key mechanism underlying functional heterogeneity. Mechanistic studies further indicate that external stimuli, such as inflammatory signals, can reshape the EV miRNA landscape, thereby modulating downstream signaling pathways in recipient cells. For example, miR‐409‐3p enriched in EVs from activated MCs has been shown to promote NF‐κB signaling and inflammatory responses in recipient cells, illustrating how stimulus‐dependent miRNA loading translates into functional effects [19]. Notably, EV‐associated miRNAs may function not only as biomarkers but also as tunable regulators of MC plasticity, offering potential therapeutic strategies, including silencing pathogenic miRNAs or restoring homeostatic regulatory networks.

Heterogeneity also exists in the EVs derived even from the same MCs. For instance, upon activation with SCF, a ligand of KIT, the human MCs line LAD2 produces two subgroups: KIT^+^ large EVs (with higher KIT) and KIT^+^ small EVs (with lower KIT). The levels of EV protein markers such as CD81, CD9, and ARF6 decrease in both EVs, suggesting that SCF stimulation may alter their protein composition. Furthermore, the KIT^+^ large EVs is associated with actin filaments and the cytoskeleton, whereas the KIT^+^ small EVs is associated with the ESCRT‐I complex, which mediates the formation of EVs [26]. Collectively, these findings indicate that MC‐EVs exhibit multi‐layered heterogeneity, arising from activation states, cargo sorting mechanisms, and EV biogenesis pathways. Such heterogeneity is further shaped by interactions with EVs from other cell types within tissue microenvironments, reinforcing the complexity of EV‐mediated signaling networks. However, whether these features are conserved across different stimuli and tissue contexts remains to be further elucidated.

### 1.2 MC‐EVs Steer the Conversation Between MCs and Other Immune Cells

MCs act as stimulators in innate immunity and as modulators and effectors in adaptive immunity, through direct cell‐cell interactions and the secretion of immunomodulatory factors [27, 28]. They function in immune activation or tolerance by acting on various T cells [29, 30, 31]. Accumulating evidence since 2020 has established EVs as key players in these communication processes (Table 2 Functional effects of exosomal components interacting with target cells).

**Table 2:** Functional effects of exosomal components interacting with target cells.

Target cells	Exosomal components	Functional effects
Dendritic cells (DCs)	HSP60/HSC70, Antigen complexes	Promotes DC maturation and enhances antigen‐presenting capacity.
ILC2 Cells	miR‐103a‐3p	Enhances IL‐5 secretion and promotes eosinophilic inflammation.
Th2 Cells	OX40L	Induces differentiation and enhances Th2‐type immune responses.
B/T Cells	MHC II, Adhesion molecules	Directly activates proliferation and cytokine secretion.

### 1.3 MC‐EVs Influence Antigen Presentation and Maturation of Dendritic Cells

MCs and dendritic cells (DCs) establish bidirectional communication not only through cytokine networks and direct membrane contacts [32], but more importantly via EVs‐mediated antigen transfer. Mechanistic studies using BMMCs reveal that endocytosed antigens (e.g., OVA) undergo proteasomal processing prior to being packaged into EVs [33]. This EV‐mediated antigen delivery is specifically mediated by molecular chaperones HSP60 and HSC70, which form stable complexes with antigens in MC‐EVs through their substrate‐binding domains. Notably, EVs from B cells or macrophages lack these HSPs (< 5% abundance compared to MC‐EVs by mass spectrometry), highlighting the unique immunogenic properties of MC‐EVs. In vivo validation shows that MC‐EV‐educated DCs migrate to splenic T cell zones within 24 h, inducing antigen‐specific IgG1/IgG2a production and IFN‐γ⁺ CD8⁺ T cell expansion [33]. This mechanism allows T cells to respond effectively even when their MHC molecules don't perfectly match, as proven by successful immune activation between mice with different genetic backgrounds (H‐2b and H‐2d strains).

Emerging evidence highlights a bidirectional regulatory axis between MC‐EVs and DC‐derived EVs (DC‐EVs). CD301b^+^ DCs strategically positioned in perivascular niches act as sentinels for blood‐borne allergens through their mannose receptors (MR). Upon allergen capture (e.g., TNP‐OVA), these DCs dynamically release antigen‐laden EVs via a VPS4‐dependent ESCRT‐III mechanism. These EVs serve as antigenic shuttles, delivering allergens to adjacent IgE‐primed MCs with > 80% degranulation efficiency (vs. 12% with free antigen). Notably, DC‐EV release is antigen‐dependent, contrasting with MCs' constitutive EV secretion in steady‐state conditions. Proteomic profiling reveals DC‐EVs are enriched in MR cytoplasmic domain fragments that retain antigen‐binding capacity, enabling sustained MC activation [34].

### 1.4 MC‐EVs Induce Lymphocyte Activation

T lymphocytes, including regulatory T cells (Tregs) and helper T cells (Th), are key protagonists in immune recognition. Th cells can differentiate into Th1 or Th2 cells, which mediate immune responses against intracellular and extracellular microorganisms, respectively [35]. MCs modulate T cell activity through multifaceted mechanisms: Antigen presentation via MHC‐II complexes to effector T cells, promoting antigen‐specific Treg expansion; Co‐stimulatory interactions mediated by MC surface molecules such as ICAM‐1 (CD54) and LFA‐1 (CD11a), which stabilize T cell adhesion and activation; OX40L‐OX40 signaling, a pathway critical for Th2 polarization [36].

Studies have shown that co‐culture of MC lines, P815 and MC/9, with spleen cells or injection of purified MC‐EVs induced blast formation, proliferation, and production of IL‐2 and IFN‐γ. These responses were predominantly Th1‐related, as IL‐4 was not detected. The molecular mechanism of activation may involve adhesion‐induced MC degranulation through LFA‐1 and ICAM‐1. The accumulation of MHC class II molecules in vesicles synthesized by MCs may enhance antigen presentation [37], although this is not yet fully understood.

Emerging studies reveal a nuanced role of MC‐EVs in Th2‐type immunity. BMMC‐derived EVs (BMMC‐EVs) were shown to synergize with IL‐4 to promote naive CD4^+^ T cell differentiation toward Th2 phenotypes, a process mediated by OX40L‐OX40 ligand‐receptor engagement, However, the in vivo relevance and quantitative contribution of EV‐associated OX40L to Th2 polarization remain debated, potentially reflecting tissue‐specific microenvironmental cue [38]. Intriguingly, EV‐mediated Th2 modulation extends beyond adaptive immunity. Type 2 innate lymphoid cells (ILC2s)—key producers of IL‐5 and IL‐13 under IL‐25/IL‐33 stimulation —exhibit selective IL‐5 upregulation when exposed to miR‐103a‐3p‐enriched EVs from human synovial MCs. This miRNA‐specific effect stems from epigenetic reprogramming of GATA3—miR‐103a‐3p suppresses PRMT5 expression, thereby reducing arginine methylation of GATA3 and potentiating its transcriptional activation of IL5 over IL13 [39].

## 2 Effects of MC‐EVs on Epithelial Cells

### 2.1 MC‐EVs Drive Mesenchymal Transformation in Epithelial Cells

The epithelial‐mesenchymal transition (EMT) is a cellular process where stationary epithelial cells morph into mobile mesenchymal phenotype, characterized by a shift from protein expressing from E‐cadherin to N‐cadherin, and subsequent changes in cell polarity, migration, and invasion capabilities [40].

In airway epithelial cells (e.g., A549), MC‐EVs drive EMT through TGF‐β1/SMAD2 signaling—surface‐bound TGF‐β1 activates sustained SMAD2 phosphorylation, upregulating EMT transcription factors (TWIST1, MMP9) while suppressing E‐cadherin and inducing N‐cadherin. Concurrently, MC‐EVs activate PI3K‐AKT and HIF‐1α pathways, synergizing to enhance metalloproteinase secretion (MMP‐2/9 activity) and cytoskeletal remodeling [41]. However, in placental trophoblasts (HTR‐8/SVneo), MC‐EVs from preeclamptic patients exert anti‐EMT effects via miR‐181a‐5p—this miRNA directly targets SNAI2, restoring E‐cadherin while inhibiting N‐cadherin/MMP‐9, ultimately impairing cell migration [42]. These opposing outcomes stem from MC heterogeneity (pro‐tumor vs. placental subtypes), signaling bias (TGF‐β1 dominance vs. miRNA‐mediated suppression), and receptor cell‐specific programming (e.g., trophoblast miR‐34c‐5p counteracts EMT attenuation), underscoring the microenvironment's pivotal role in dictating EV functionality.

### 2.2 Effects of MC‐EVs on Epithelial Cell Barrier

MCs can be activated under physiological stresses in some pathogenic disorders, leading to increased permeability of the intestinal epithelial barrier. The degree of MC infiltration is associated with changes in epithelial barrier permeability [43, 44]

MC‐EVs demonstrate divergent roles in modulating intestinal barrier integrity, contingent upon their cellular origin and pathophysiological context. Treatment with MC‐EVs from the HMC‐1 cell line significantly compromises epithelial barrier function in intestinal cell models (NCM460, HT‐29, and CaCO2), a process mediated by miR‐223 enrichment within these vesicles. This miRNA suppresses critical tight junction components, including zonula occludens‐1 (ZO‐1), occludin (OCLN), and claudin‐8 (CLDN8), thereby destabilizing intercellular junctions [45]. Conversely, preliminary evidence suggests that MC‐EVs isolated from duodenal mucosa—or those derived from eosinophils—may paradoxically enhance epithelial integrity and mitigate functional dyspepsia in rodent models. However, these protective effects lack mechanistic validation, with no delineation of cargo‐specific contributions (e.g., anti‐inflammatory miRNAs or reparative proteins) or receptor‐cell signaling pathways [46].

### 2.3 MC‐EVs Mediate Cellular Interactions in Tumors

MCs infiltration is observed in various tumor types [10]. These cells are recruited to the tumor microenvironment (TME) via the interaction of stem cell factor (SCF) with its receptor KIT (CD117). However, their role in tumor progression remains controversial [47]. MCs promote tumor angiogenesis by releasing pro‐angiogenic factors such as histamine, heparin, and tryptase [10]. Elevated levels of tryptase‐positive MCs are frequently detected in solid tumors, including melanoma, colorectal cancer (CRC), and gastric cancer [48].

EVs are abundant in the tumor microenvironment, attuning the behaviors of tumor cells [49]. In a study, it was demonstrated that EVs derived from BMMC have an impact on the proliferation, migration and invasion of mouse hepatoma cell Hepa1‐6 [49]. Additionally, when transferred protein KIT via MC‐EVs, lung adenocarcinoma cells displayed an increase in cyclin D1 expression, leading to accelerated cell proliferation [50]. On the other hand, lung adenocarcinoma cells also produce EVs carrying SCF, which activate MCs to release tryptase. This in turn accelerates the proliferation and migration of endothelial cells, fostering angiogenesis [51].

Studies on the duty of MC‐EVs in melanoma are incompatible. EVs originating from antigen‐stimulated RBL2H3 MCs (Active‐MC‐EVs) can boost the expression of CCL2 (MCP1) in melanoma cells and lung macrophages, thereby increasing the carcinogenic and metastatic potential of melanoma cells [52]. This action is reliant on miR‐154‐5p, which is also plentiful in Active‐MC‐EVs. In contrast, Emerging evidence highlights the unconventional nuclear‐targeting activity of mast cell (MC)‐derived tryptase in melanoma. Melanoma cells secrete DNA‐coated extracellular vesicles (EVs) that selectively capture tryptase released by neighboring MCs. Following endocytosis, tryptase translocates to the nucleus, where it induces dual disruptions: (1) epigenetic remodeling through histone cleavage, silencing oncogenes such as EGR1, and (2) structural damage to the nuclear envelope via degradation of lamina‐associated proteins, ultimately triggering irreversible cell cycle arrest [53].

Therefore, it may be possible to propose a mechanism whereby the immune system, represented by MC, has the ability to hijack tumor cell‐derived exosomes for anti‐tumor purposes, since the number and variety of EVs increased significantly after MC degranulation, drug therapy that inhibits MCs degranulation may affect the release of MC ‐EVs and the inflammatory outcome [24].

### 2.4 EVs From Neoplastic MCs Affect the Progression of Mastocytosis

Mastocytosis is a heterogeneous group of hematologic neoplasms characterized by the increased production and accumulation of clonal MCs. As the disease progresses, additional affected organs emerge, including the liver (hepatomegaly), spleen (splenomegaly), and bones (osteoporosis), among others [54].

Kim, DK observed that systemic mastocytosis patients, in comparison to healthy controls, exhibited a higher concentration of serum EVs, which contained MC proteins, such as KIT, FceRI, MRGPRX2, and tryptase, but not granule substances like histamine, heparin, or prohibitin. This suggests that malignant MCs in a resting state release substantial EVs in mastocytosis, which are positively correlated with hepatosplenomegaly. Furthermore, these EVs successfully deliver KIT into a human stellate cell, stimulating proliferation, cytokine production, and differentiation ‐ processes linked to liver pathology. This effect can be tempered by KIT inhibition or neutralization, and is replicated by enforced expression of KIT or a constitutively active D816V‐KIT, a gain‐of‐function mutation associated with mastocytosis. Additionally, these researchers discovered that EVs rising from mastocytosis also disrupt osteoblast maturation, negatively affecting trabecular bone volume and microarchitecture [55]. These effects are mediated by the delivery of miRNA‐30a and miRNA‐23a through the EVs released from neoplastic MCs, which target osteogenic transcription factors RUNX2 and SMAD1/5.

In short, EVs from neoplastic MCs hold theranostic potential for mastocytosis.

### 2.5 MC‐EVs in Diverse Tissue Microenvironments

Beyond classical immune and epithelial contexts, MC‐EVs exert regulatory functions across diverse tissue microenvironments, where they interact with multiple cell types to form dynamic intercellular communication networks.

### 2.6 MC‐EVs in the Central Nervous System

In the central nervous system, MC‐EVs contribute to neuroinflammatory processes and blood‐brain barrier regulation, highlighting their role in modulating brain homeostasis and disease progression [56] [57]. A recent study [58] conducted a detailed analysis of cerebral malaria and found that intravenously injected MC‐EVs (Rest‐MC‐EVs from murine MC P815) into a mouse model of experimental cerebral malaria aggravated brain vascular endothelial activation and disrupted the blood‐brain barrier. In vitro experiments using brain microvascular endothelial cells showed that treatment with P815 cell‐derived EVs resulted in decreased mRNA levels of Ang‐1, ZO‐1, and Claudin‐5, and increased Ang‐2, CCL2, CXCL1, and CXCL9. These findings suggest that MC‐EVs can compromise the integrity of the blood‐brain barrier and intensify brain histopathological damage. Further evidence from another study shows that EVs (Active‐MC‐EVs) from Lipopolysaccharide‐stimulated P815 cell enhance cell migration, activation of microglia cells, and upregulation of CD86, IL‐1β, IL‐6 and TNF‐α. This outcome is dependent on the EV‐delivery of miR‐409‐3p, which activates the NF‐κB pathway [19]. These findings suggest that MC‐EVs can effectively penetrate the blood‐brain barrier, reaching the central nervous system from the peripheral circulation, and thus represent potential therapeutic targets and drug delivery vehicles for brain disorders.

### 2.7 MC‐EVs in the Bone Marrow Niche

In the bone marrow niche, EVs from multiple sources—including MCs, MSCs, fibroblasts, and hematopoietic cells—coexist and interact, forming a dynamic communication network rather than isolated entities [59]. This interplay modulates niche homeostasis but underscores the need for multi‐source EV profiling to mitigate heterogeneity‐related risks in clinical settings.

In particular, MC‐EVs interact with MSCs by delivering bioactive cargos, particularly surface‐associated TGFβ‐1, which promotes MSC migration and migratory phenotypic remodeling through SMAD‐dependent signaling [60]. Given the central role of MSCs in maintaining niche integrity, such EV‐mediated regulation may indirectly influence HSC maintenance and differentiation. In addition to MSCs, HSCs are highly responsive to microenvironmental cues conveyed by EVs [5]. Consistent with this, EVs derived from bone marrow MSCs of different developmental origins have been shown to differentially support ex vivo expansion of umbilical cord blood hematopoietic stem and progenitor cells, further indicating that stromal cell‐derived EV heterogeneity can directly influence hematopoietic cell behavior [61],

Beyond MSCs, fibroblasts are key stromal components of the bone marrow niche that regulate extracellular matrix organization and local signaling [59]. Mast cell‐derived EVs may be taken up by fibroblasts and promote matrix‐remodeling activity, which could alter stromal cues, including CXCL12‐dependent niche regulation [62], and thereby indirectly influence HSC maintenance and quiescence [6, 7]. Although direct evidence of MC‐EV–HSC interactions remains limited, current findings suggest that MC‐EVs may indirectly influence HSC fate through stromal cell remodeling and niche reprogramming.

Collectively, MC‐EVs orchestrate a lipid‐bilayered regulatory network with MSCs, HSCs, fibroblasts, and other immune cells, fine‐tuning bone marrow homeostasis. Dysregulation of these interactions may contribute to diseases like leukemia, warranting targeted EV interventions.

### 2.8 Clinical Potential of MC‐EVs in Allergic Diseases

The distinct role of MC‐EVs in diseases, particularly their contested impact on allergic reactions, is a significant point of discussion within the broader acknowledgment of the importance of EVs in immune regulation and therapy.

In a recent study on asthma, researchers discovered that upon activation of BMMCs by allergens leads to the release of EVs containing FcεRI. These EVs have the ability to bind and counteract free IgE in the bloodstream, consequently restraining MC activation by lowering its concentration [63]. This finding suggests a potential therapeutic approach for managing MCs hyperactivity in allergic conditions. Conversely, additional evidence reinforces the beneficial regulatory function of MC‐ EVs [33, 37, 64]. In one investigation [64], after stimulation by an allergen, FcεRI complex was found in MC‐EVs. When IgE or antigen attaches to FcεRI, these MC‐EVs carry receptors, IgE, and antigens, working together with soluble antigens to activate additional MCs. This interaction amplifies the inflammatory response.

The RNA transported via MC‐EVs holds significant sway over various facets of human health and disease. MC‐EVs are abundant in miR103a‐3p and miR‐142‐3p, both of which actively encourage MC degranulation via the FcεRI receptor [18, 25]. The tRNA‐derived fragment tRF‐Leu‐AAG‐001 detected in leukocyte‐derived EVs from individuals with endometriosis originates specifically from MC situated within ectopic tissues. These findings underscore the potential therapeutic application of engineered MC‐EVs.

## 3 Future Directions and Conclusions

**Figure 1:**
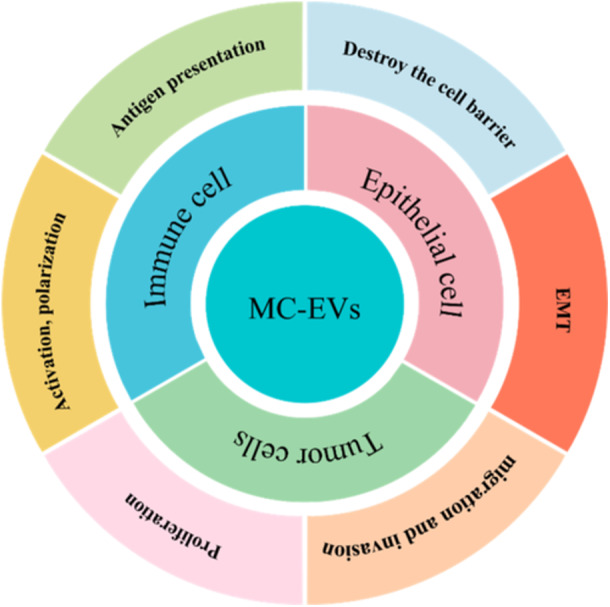
MC‐EVs mediate intercellular communication in both physiological and pathological processes. MC‐EVs facilitate communication between cells by amplifying the antigen presentation of dendritic cells, intensifying T cell activation, and guiding macrophage polarization towards M2. MC‐EVs instigate numerous signaling events within epithelial cells, encouraging an epithelial‐to‐mesenchymal transition (EMT) for cell reprogramming while simultaneously disrupting cell barriers by inhibiting tight junction proteins and elevating epithelial permeability. Across cancer progression, MC‐EVs interact within the tumor microenvironment (TME), impacting cancer cell proliferation and migration. Additionally, MC‐EVs target other receptor cells such as endothelial cells, mesenchymal stem cells (MSCs), and nerve cells.

The following is a summary of the whole review, which contains the structure of the paper. figure 1

The field of EVs related with MCs is rapidly evolving, and there are several important areas that warrant further research. First, it is essential to explore the factors contributing to the heterogeneity of MC‐EVs under varying conditions. The heterogeneity of EVs, arising from multiple cellular sources rather than a singular origin, must be evaluated within complex tissue microenvironments where EVs from coexisting cell types interact dynamically. This diversity enhances therapeutic potential but poses challenges for clinical trials, such as variability in efficacy and off‐target effects. Emerging technologies like single‐vesicle proteomics, single‐cell transcriptomics, and spatial omics can dissect MC‐EV heterogeneity, enabling precise isolation and engineering for targeted therapies in inflammation and cancer.

Second, it is imperative to understand the specific molecular components within MC‐EVs to discern their effects on distinct cell types. Analytical techniques such as proteomic, genomic, and lipidomic analyses can be employed to identify these key elements. This insight could expedite the development of tailored therapies by manipulating the cargo of MC‐EVs for targeted therapeutic applications. Future studies should delineate MC‐EV cargo delivery mechanisms and downstream signaling cascades (e.g., NF‐κB, TGF‐β, and Wnt pathways) to explain context‐specific effects on recipient cells. Defining these pathways could underpin precision therapies, including EV‐based drug delivery for inflammatory disorders, tumor microenvironments, and hematopoietic regeneration, while addressing challenges like pathway crosstalk in heterogeneous settings.

Third, decipher the mechanisms of MC‐EV uptake by recipient cells to fully understand their mode of action. Fluctuations in membrane molecules on both MC‐EVs and recipient cells can alter the endocytosis process and subsequent cellular responses. This, in turn, can potentially impact the selective uptake of MC‐EVs by recipient cells and the resulting functional effects.A more detailed understanding of cargo delivery and downstream signaling pathways will also help explain how MC‐EVs exert distinct biological effects in different recipient cells.

Lastly, the development of therapeutic strategies based on MC‐EVs itself shows great promise. MC‐EVs have demonstrated potential as a tool for vaccine development and delivery, as they can modulate immune responses, reduce IgE levels, and enhance antigen presentation in the context of allergies. Modulating MC‐EV release, cargo composition, and uptake by recipient cells through engineering and targeting approaches offer potential therapeutic interventions. However, developing these strategies requires a deep understanding of MC‐EV biology and their interactions with recipient cells. Moreover, defining the molecular signaling pathways regulated by MC‐EVs may provide an important basis for precision therapeutic targeting in inflammatory, neoplastic, and hematopoietic disorders.

In conclusion, MC‐EVs exhibit diverse characteristics and are essential for intercellular communication, impacting immune responses, epithelial cell behavior, and other cellular functions.

## Author Contributions

**Bingqi Zhang:** methodology, writing – original draft, funding acquisition, conceptualization. **Yueshan Sun:** writing – review and editing, funding acquisition. **Tao Jiang:** data curation, investigation. **Runmin Long:** Investigation. **Yuanbiao Guo:** writing – review and editing, funding acquisition.

## Conflicts of Interest

The authors declare no conflicts of interest.

## Data Availability

No new data were created or analyzed in this study. Data sharing is not applicable to this article.

## References

[1] G. Sammarco , G. Varricchi , V. Ferraro , et al., “Mast Cells, Angiogenesis and Lymphangiogenesis in Human Gastric Cancer,” International Journal of Molecular Sciences 20 (2019): 2106. 31035644 10.3390/ijms20092106 PMC6540185

[2] S. Pal , S. Nath , C. J. Meininger , and A. A. Gashev , “Emerging Roles of Mast Cells in the Regulation of Lymphatic Immuno‐Physiology,” Frontiers in Immunology 11 (2020): 1234. 32625213 10.3389/fimmu.2020.01234 PMC7311670

[3] A. M. Irani , T. R. Bradford , C. L. Kepley , N. M. Schechter , and L. B. Schwartz , “Detection of MCT and MCTC Types of Human Mast Cells by Immunohistochemistry Using New Monoclonal Anti‐Tryptase and Anti‐Chymase Antibodies,” Journal of Histochemistry & Cytochemistry 37 (1989): 1509–1515. 2674273 10.1177/37.10.2674273

[4] L. Kaltenbach , P. Martzloff , S. K. Bambach , et al., “Slow Integrin‐Dependent Migration Organizes Networks of Tissue‐Resident Mast Cells,” Nature Immunology 24 (2023): 915–924. 37081147 10.1038/s41590-023-01493-2 PMC10232366

[5] J. T. Butler , S. Abdelhamed , and P. Kurre , “Extracellular Vesicles in the Hematopoietic Microenvironment,” Haematologica 103 (2018): 382–394. 29439185 10.3324/haematol.2017.183335 PMC5830368

[6] Y. Taketomi , T. Higashi , K. Kano , et al., “Lipid‐Orchestrated Paracrine Circuit Coordinates Mast Cell Maturation and Anaphylaxis Through Functional Interaction With Fibroblasts,” Immunity 57 (2024): 1828–1847.e11. 39002541 10.1016/j.immuni.2024.06.012

[7] S. Muntión , T. L. Ramos , M. Diez‐Campelo , et al., “Microvesicles From Mesenchymal Stromal Cells are Involved in HPC‐Microenvironment Crosstalk in Myelodysplastic Patients,” PLoS One 11 (2016): e0146722. 26836120 10.1371/journal.pone.0146722 PMC4737489

[8] P. Kolkhir , D. Elieh‐Ali‐Komi , M. Metz , F. Siebenhaar , and M. Maurer , “Understanding Human Mast Cells: Lesson From Therapies for Allergic and Non‐Allergic Diseases,” Nature Reviews Immunology 22 (2022): 294–308. 10.1038/s41577-021-00622-y 34611316

[9] K. Katsoulis‐Dimitriou , J. Kotrba , M. Voss , J. Dudeck , and A. Dudeck , “Mast Cell Functions Linking Innate Sensing to Adaptive Immunity,” Cells 9 (2020): 2538. 33255519 10.3390/cells9122538 PMC7761480

[10] D. E. A. Komi and F. A. Redegeld , “Role of Mast Cells in Shaping the Tumor Microenvironment,” Clinical Reviews in Allergy & Immunology 58 (2020): 313–325. 31256327 10.1007/s12016-019-08753-w PMC7244463

[11] S. Cheng , Z. Li , R. Gao , et al., “A Pan‐Cancer Single‐Cell Transcriptional Atlas of Tumor Infiltrating Myeloid Cells,” Cell 184 (2021): 792–809.e23. 33545035 10.1016/j.cell.2021.01.010

[12] I. Shefler , P. Salamon , T. Zitman‐Gal , and Y. A. Mekori , “Tumor‐Derived Extracellular Vesicles Induce CCL18 Production by Mast Cells: A Possible Link to Angiogenesis,” Cells 11 (2022): 353. 35159163 10.3390/cells11030353 PMC8834361

[13] A. Benito‐Martin , L. Nogués , M. Hergueta‐Redondo , et al., “Mast Cells Impair Melanoma Cell Homing and Metastasis by Inhibiting HMGA1 Secretion,” Immunology 168 (2023): 362–373. 36352838 10.1111/imm.13604

[14] P. Salamon , Y. A. Mekori , and I. Shefler , “Lung Cancer‐Derived Extracellular Vesicles: A Possible Mediator of Mast Cell Activation in the Tumor Microenvironment,” Cancer Immunology, Immunotherapy 69 (2020): 373–381. 31897659 10.1007/s00262-019-02459-w PMC11027796

[15] O. Klein and R. Sagi‐Eisenberg , “Anaphylactic Degranulation of Mast Cells: Focus on Compound Exocytosis,” Journal of Immunology Research 2019 (2019): 9542656. 31011586 10.1155/2019/9542656 PMC6442490

[16] Y. Liang , S. Huang , L. Qiao , et al., “Characterization of Protein, Long Noncoding RNA and MicroRNA Signatures in Extracellular Vesicles Derived From Resting and Degranulated Mast Cells,” Journal of Extracellular Vesicles 9 (2020): 1697583. 31853339 10.1080/20013078.2019.1697583 PMC6913652

[17] T. Groot Kormelink , G. J. A. Arkesteijn , C. H. A. van de Lest , et al., “Mast Cell Degranulation is Accompanied by the Release of a Selective Subset of Extracellular Vesicles That Contain Mast Cell‐Specific Proteases,” Journal of Immunology 197 (2016): 3382–3392. 10.4049/jimmunol.1600614 27619994

[18] S. Toyoshima , T. Sakamoto‐Sasaki , Y. Kurosawa , et al., “miR103a‐3p in Extracellular Vesicles From FcεRI‐Aggregated Human Mast Cells Enhances IL‐5 Production by Group 2 Innate Lymphoid Cells,” Journal of Allergy and Clinical Immunology 147 (2021): 1878–1891. 33465368 10.1016/j.jaci.2021.01.002

[19] L. Hu , L. Si , X. Dai , et al., “Exosomal miR‐409‐3p Secreted From Activated Mast Cells Promotes Microglial Migration, Activation and Neuroinflammation by Targeting Nr4a2 to Activate the NF‐κB Pathway,” Journal of Neuroinflammation 18 (2021): 68. 33750404 10.1186/s12974-021-02110-5 PMC7945321

[20] M. Tsai , P. Valent , and S. J. Galli , “KIT as a Master Regulator of the Mast Cell Lineage,” Journal of Allergy and Clinical Immunology 149 (2022): 1845–1854. 35469840 10.1016/j.jaci.2022.04.012 PMC9177781

[21] Y. Nagata and R. Suzuki , “FcεRI: A Master Regulator of Mast Cell Functions,” Cells 11 (2022): 622. 35203273 10.3390/cells11040622 PMC8870323

[22] T. E. Frederick , J. N. Chebukati , C. E. Mair , P. C. Goff , and G. E. Fanucci , “Bis(Monoacylglycero)Phosphate Forms Stable Small Lamellar Vesicle Structures: Insights Into Vesicular Body Formation in Endosomes,” Biophysical Journal 96 (2009): 1847–1855. 19254543 10.1016/j.bpj.2008.12.3892 PMC2717297

[23] T. Skotland , N. P. Hessvik , K. Sandvig , and A. Llorente , “Exosomal Lipid Composition and the Role of Ether Lipids and Phosphoinositides in Exosome Biology,” Journal of Lipid Research 60 (2019): 9–18. 30076207 10.1194/jlr.R084343 PMC6314266

[24] M. Lecce , R. Molfetta , N. D. Milito , A. Santoni , and R. Paolini , “FcεRI Signaling in the Modulation of Allergic Response: Role of Mast Cell‐Derived Exosomes,” International Journal of Molecular Sciences 21 (2020): 5464. 32751734 10.3390/ijms21155464 PMC7432241

[25] Y. Yamada , K. Kosaka , T. Miya zawa , K. Kurata‐Miura , and T. Yoshida , “MiR‐142‐3p Enhances FcεRI‐Mediated Degranulation in Mast Cells,” Biochemical and Biophysical Research Communications 443 (2014): 980–986. 24361879 10.1016/j.bbrc.2013.12.078

[26] A. Pfeiffer , J. D. Petersen , G. H. Falduto , et al., “Selective Immunocapture Reveals Neoplastic Human Mast Cells Secrete Distinct Microvesicle‐ and Exosome‐Like Populations of KIT‐Containing Extracellular Vesicles,” Journal of Extracellular Vesicles 11 (2022): e12272. 36239715 10.1002/jev2.12272 PMC9838129

[27] J. B. Huppa and M. M. Davis , “T‐Cell‐Antigen Recognition and the Immunological Synapse,” Nature Reviews Immunology 3 (2003): 973–983. 10.1038/nri1245 14647479

[28] R. Joulia , N. Gaudenzio , M. Rodrigues , et al., “Mast Cells Form Antibody‐Dependent Degranulatory Synapse for Dedicated Secretion and Defence,” Nature Communications 6 (2015): 6174. 10.1038/ncomms7174 25629393

[29] S. Bulfone‐Paus and R. Bahri , “Mast Cells as Regulators of T Cell Responses,” Frontiers in Immunology 6 (2015): 394. 26300882 10.3389/fimmu.2015.00394 PMC4528181

[30] L. F. Lu , E. F. Lind , D. C. Gondek , et al., “Mast Cells are Essential Intermediaries in Regulatory T‐Cell Tolerance,” Nature 442 (2006): 997–1002. 16921386 10.1038/nature05010

[31] D. B. Leveson‐Gower , E. I. Sega , J. Kalesnikoff , et al., “Mast Cells Suppress Murine GVHD in a Mechanism Independent of CD4+CD25+ Regulatory T Cells,” Blood 122 (2013): 3659–3665. 24030387 10.1182/blood-2013-08-519157 PMC3837515

[32] K. Kalkusova , S. Smite , E. Darras , et al., “Mast Cells and Dendritic Cells as Cellular Immune Checkpoints in Immunotherapy of Solid Tumors,” International Journal of Molecular Sciences 23 (2022): 11080. 36232398 10.3390/ijms231911080 PMC9569882

[33] D. Skokos , H. G. Botros , C. Demeure , et al., “Mast Cell‐Derived Exosomes Induce Phenotypic and Functional Maturation of Dendritic Cells and Elicit Specific Immune Responses In Vivo,” Journal of Immunology 170 (2003): 3037–3045. 10.4049/jimmunol.170.6.3037 12626558

[34] H. W. Choi , J. Suwanpradid , I. H. Kim , et al., “Perivascular Dendritic Cells Elicit Anaphylaxis by Relaying Allergens to Mast Cells via Microvesicles,” Science 362 (2018): eaao0666. 30409859 10.1126/science.aao0666 PMC6376486

[35] J. Zhu , “T Helper Cell Differentiation, Heterogeneity, and Plasticity,” Cold Spring Harbor Perspectives in Biology 10 (2018): a030338. 28847903 10.1101/cshperspect.a030338 PMC6169815

[36] A. Y. Hershko and J. Rivera , “Mast Cell and T Cell Communication; Amplification and Control of Adaptive Immunity,” Immunology Letters 128 (2010): 98–104. 19900479 10.1016/j.imlet.2009.10.013 PMC2823825

[37] D. Skokos , S. Le Panse , I. Villa , et al., “Mast Cell‐Dependent B and T Lymphocyte Activation is Mediated by the Secretion of Immunologically Active Exosomes,” Journal of Immunology 166 (2001): 868–876. 10.4049/jimmunol.166.2.868 11145662

[38] F. Li , Y. Wang , L. Lin , et al., “Mast Cell‐Derived Exosomes Promote th2 Cell Differentiation via OX40L‐OX40 Ligation,” Journal of Immunology Research 2016 (2016): 3623898. 27066504 10.1155/2016/3623898 PMC4811108

[39] J. Qiu , J. Zhang , Y. Ji , et al., “Tissue Signals Imprint Aiolos Expression in ILC2s to Modulate Type 2 Immunity,” Mucosal Immunology 14 (2021): 1306–1322. 34349237 10.1038/s41385-021-00431-5 PMC8528704

[40] B. Bakir , A. M. Chiarella , J. R. Pitarresi , and A. K. Rustgi , “EMT, MET, Plasticity, and Tumor Metastasis,” Trends in Cell Biology 30 (2020): 764–776. 32800658 10.1016/j.tcb.2020.07.003 PMC7647095

[41] Y. Yin , G. V. Shelke , C. Lässer , H. Brismar , and J. Lötvall , “Extracellular Vesicles From Mast Cells Induce Mesenchymal Transition in Airway Epithelial Cells,” Respiratory Research 21 (2020): 101. 32357878 10.1186/s12931-020-01346-8 PMC7193353

[42] Y. Wang and A. Chen , “Mast Cell‐Derived Exosomal miR‐181a‐5p Modulated Trophoblast Cell Viability, Migration, and Invasion via YY1/MMP‐9 Axis,” Journal of Clinical Laboratory Analysis 36 (2022): e24549. 35698293 10.1002/jcla.24549 PMC9280008

[43] D. Kempuraj , S. Mentor , R. Thangavel , et al., “Mast Cells in Stress, Pain, Blood‐Brain Barrier, Neuroinflammation and Alzheimer's Disease,” Frontiers in Cellular Neuroscience 13 (2019): 54. 30837843 10.3389/fncel.2019.00054 PMC6389675

[44] G. Traina , “The Role of Mast Cells in the Gut and Brain,” Journal of Integrative Neuroscience 20 (2021): 185–196. 33834706 10.31083/j.jin.2021.01.313

[45] M. Li , J. Zhao , M. Cao , et al., “Mast Cells‐Derived MiR‐223 Destroys Intestinal Barrier Function by Inhibition of CLDN8 Expression in Intestinal Epithelial Cells,” Biological Research 53 (2020): 12. 32209121 10.1186/s40659-020-00279-2 PMC7092522

[46] J. Wang , S. Gu , and B. Qin , “Eosinophil and Mast Cell‐Derived Exosomes Promote Integrity of Intestinal Mucosa via the NEAT1/miR‐211‐5p/glial Cell Line‐Derived Neurotrophic Factor Axis in Duodenum,” Environmental Toxicology 38 (2023): 2595–2607. 37466184 10.1002/tox.23895

[47] J. S. Dahlin , M. Ekoff , J. Grootens , et al., “KIT Signaling is Dispensable for Human Mast Cell Progenitor Development,” Blood 130 (2017): 1785–1794. 28790106 10.1182/blood-2017-03-773374 PMC5659818

[48] G. Paolino , P. Corsetti , E. Moliterni , et al., “Mast Cells and Cancer,” Giornale Italiano di Dermatologia e Venereologia 154 (2019): 650–668. 29192477 10.23736/S0392-0488.17.05818-7

[49] X. D. Wang , M. Q. Yang , B. Liu , et al., “Effects of Bone Marrow‐Derived Mast Cell Exosomes on Proliferation, Migration and Invasion of Hepatocellular Carcinoma Hepa1‐6 Cells,” Acta Universitatis Medicinalis Anhui (安徽医科大学学报) 55, no. 9 (2020): 1394–1399.

[50] H. Xiao , C. Lässer , G. V. Shelke , et al., “Mast Cell Exosomes Promote Lung Adenocarcinoma Cell Proliferation–Role of KIT‐Stem Cell Factor Signaling,” Cell Communication and Signaling 12 (2014): 64. 25311367 10.1186/s12964-014-0064-8 PMC4206705

[51] H. Xiao , M. He , G. Xie , et al., “The Release of Tryptase From Mast Cells Promote Tumor Cell Metastasis via Exosomes,” BMC Cancer 19 (2019): 1015. 31664930 10.1186/s12885-019-6203-2 PMC6819443

[52] M. Kim , H. Jo , Y. Kwon , et al., “MiR‐154‐5p‐MCP1 Axis Regulates Allergic Inflammation by Mediating Cellular Interactions,” Frontiers in Immunology 12 (2021): 663726. 34135893 10.3389/fimmu.2021.663726 PMC8201518

[53] F. Rabelo Melo , S. Santosh Martin , C. P. Sommerhoff , and G. Pejler , “Exosome‐Mediated Uptake of Mast Cell Tryptase into the Nucleus of Melanoma Cells: A Novel Axis for Regulating Tumor Cell Proliferation and Gene Expression,” Cell Death & Disease 10 (2019): 659. 31506436 10.1038/s41419-019-1879-4 PMC6736983

[54] J. Gotlib , A. Pardanani , C. Akin , et al., “International Working Group‐Myeloproliferative Neoplasms Research and Treatment (IWG‐MRT) & European Competence Network on Mastocytosis (ECNM) Consensus Response Criteria in Advanced Systemic Mastocytosis,” Blood 121 (2013): 2393–2401. 23325841 10.1182/blood-2012-09-458521 PMC3612852

[55] D. K. Kim , G. Bandara , Y. E. Cho , et al., “Mastocytosis‐Derived Extracellular Vesicles Deliver miR‐23a and miR‐30a Into Pre‐Osteoblasts and Prevent Osteoblastogenesis and Bone Formation,” Nature Communications 12 (2021): 2527. 10.1038/s41467-021-22754-4 PMC8100305 33953168

[56] P. Esposito , D. Gheorghe , K. Kandere , et al., “Acute Stress Increases Permeability of the Blood‐Brain‐Barrier Through Activation of Brain Mast Cells,” Brain Research 888 (2001): 117–127. 11146058 10.1016/s0006-8993(00)03026-2

[57] H. Tran , A. Mittal , V. Sagi , et al., “Mast Cells Induce Blood Brain Barrier Damage in SCD by Causing Endoplasmic Reticulum Stress in the Endothelium,” Frontiers in Cellular Neuroscience 13 (2019): 56. 30837844 10.3389/fncel.2019.00056 PMC6389721

[58] K. Huang , L. Huang , X. Zhang , et al., “Mast Cells‐Derived Exosomes Worsen the Development of Experimental Cerebral Malaria,” Acta Tropica 224 (2021): 106145. 34562426 10.1016/j.actatropica.2021.106145

[59] Z. Huang , Z. Iqbal , Z. Zhao , et al., “Cellular Crosstalk in the Bone Marrow Niche,” Journal of Translational Medicine 22 (2024): 1096. 39627858 10.1186/s12967-024-05900-6 PMC11613879

[60] G. V. Shelke , Y. Yin , S. C. Jang , et al., “Endosomal Signalling via Exosome Surface TGFβ‐1,” Journal of Extracellular Vesicles 8 (2019): 1650458. 31595182 10.1080/20013078.2019.1650458 PMC6764367

[61] C. A. Ghebes , J. Morhayim , M. Kleijer , et al., “Extracellular Vesicles Derived From Adult and Fetal Bone Marrow Mesenchymal Stromal Cells Differentially Promote Ex Vivo Expansion of Hematopoietic Stem and Progenitor Cells,” Frontiers in Bioengineering and Biotechnology 9 (2021): 640419. 33718342 10.3389/fbioe.2021.640419 PMC7947881

[62] P. Scala , B. Serio , and V. Giudice , “3D In Vitro Models of the Bone Marrow Niche,” ACS Biomaterials Science & Engineering 12 (2026): 110–127. 41384609 10.1021/acsbiomaterials.5c01421 PMC12801197

[63] G. Xie , H. Yang , X. Peng , et al., “Mast Cell Exosomes can Suppress Allergic Reactions by Binding to IgE,” Journal of Allergy and Clinical Immunology 141 (2018): 788–791. 28916187 10.1016/j.jaci.2017.07.040

[64] R. Molfetta , M. Lecce , L. Quatrini , et al., “Immune Complexes Exposed on Mast Cell‐Derived Nanovesicles Amplify Allergic Inflammation,” Allergy 75 (2020): 1260–1263. 31713871 10.1111/all.14103

